# The RhoA-Rok-Myosin II Pathway is Involved in Extracellular Matrix-Mediated Regulation of Prolactin Signaling in Mammary Epithelial Cells

**DOI:** 10.1002/jcp.22886

**Published:** 2011-06-15

**Authors:** Jyun-Yi Du, Meng-Chi Chen, Tsai-Ching Hsu, Jen-Hsing Wang, Lisa Brackenbury, Ting-Hui Lin, Yi-Ying Wu, Zhihong Yang, Charles H Streuli, Yi-Ju Lee

**Affiliations:** 1 Institute of Microbiology and Immunology, Chung Shan Medical UniversityTaichung, Taiwan, ROC; 2 Department of Obstetrics and Gynecology, Antai Tian-Sheng Memorial HospitalPingtung, Taiwan, ROC; 3 Wellcome Trust Centre for Cell-Matrix Research, Faculty of Life Sciences, and Manchester Breast Centre, University of ManchesterManchester, England, UK; 4 Department of Biomedical Sciences, Chung Shan Medical UniversityTaichung, Taiwan, ROC; 5 Department of Medical Laboratory Science and Biotechnology, China Medical University and HospitalTaichung, Taiwan, ROC; 6 Vascular Biology, Institute of Physiology, University of FribourgFribourg, Switzerland; 7 Department of Medical Research, Chung Shan Medical University HospitalTaichung, Taiwan, ROC

## Abstract

In mammary epithelial cells (MECs), prolactin-induced signaling and gene expression requires integrin-mediated cell adhesion to basement membrane (BM). In the absence of proper cell–BM interactions, for example, culturing cells on collagen-coated plastic dishes, signal propagation is substantially impaired. Here we demonstrate that the RhoA-Rok-myosin II pathway accounts for the ineffectiveness of prolactin signaling in MECs cultured on collagen I. Under these culture conditions, the RhoA pathway is activated, leading to downregulation of prolactin receptor expression and reduced prolactin signaling. Enforced activation of RhoA in MECs cultured on BM suppresses prolactin receptor levels, and prevents prolactin-induced Stat5 tyrosine phosphorylation and β-casein expression. Overexpression of dominant negative RhoA in MECs cultured on collagen I, or inhibiting Rok activity, increases prolactin receptor expression, and enhances prolactin signaling. In addition, inhibition of myosin II ATPase activity by blebbistatin also exerts a beneficial effect on prolactin receptor expression and prolactin signaling, suggesting that tension exerted by the collagen substratum, in collaboration with the RhoA-Rok-myosin II pathway, contributes to the failure of prolactin signaling. Furthermore, MECs cultured on laminin-coated plastic have similar morphology and response to prolactin as those cultured on collagen I. They display high levels of RhoA activity and are inefficient in prolactin signaling, stressing the importance of matrix stiffness in signal transduction. Our results reveal that RhoA has a central role in determining the fate decisions of MECs in response to cell–matrix interactions. J. Cell. Physiol. 227: 1553–1560, 2012. © 2011 Wiley Periodicals, Inc.

Extracellular matrix (ECM) influences cell behavior by signaling through integrins (Streuli, 2009). Rho GTPases are activated in response to integrin engagement, triggering cytoskeleton reorganization and signal propagation to govern a number of biological events, such as cell cycle progression, cytokinesis, morphogenesis, and migration (Heasman and Ridley, 2008). These diversified functions are accomplished by activation of an assortment of effectors downstream of Rho GTPases. In the case of RhoA, many effectors have been identified, including Rho kinase (Rok). Rok activates myosin II by phosphorylating myosin light chain and myosin light chain phosphatase. This promotes stress fiber formation and cellular contractility, and thus modulates cell–cell adhesion, cell–matrix adhesion, migration, and polarity (Vicente-Manzanares et al., 2009). In addition to stimulating myosin-related contraction, Rok also regulates phagocytosis, apoptosis, differentiation, and cell size (Riento and Ridley, 2003).

Like all epithelial cells, mammary epithelial cells (MECs) contact basement membrane (BM) in vivo. The 3D acinar morphology and functional differentiation of MECs can be recapitulated in vitro by culturing cells on a reconstituted BM matrix. By contrast, MECs grown on standard tissue culture plastic or on dishes coated with collagen I, which is the dominant stromal matrix component in mammary glands, form monolayers and are unresponsive to lactogenic hormones (prolactin, insulin, and hydrocortisone)(Streuli et al., 1991). Several lines of evidence reveal that laminin, the major component of BM, instructs mammary morphogenesis and functions by signaling through its receptors, dystroglycan and β1 integrin (Streuli et al., 1995; Naylor et al., 2005; Leonoudakis et al., 2010). The former receptor helps to anchor the BM to the cell surface, whereas the latter conveys signal into cells. Ablation of either dystroglycan or β1 integrin results in disruption of tissue architecture and inhibition of β-casein expression. Recent studies have elucidated that integrin-linked kinase (ILK) links β1 integrin to Rac1, facilitating prolactin-induced Jak2-Stat5 pathway and, ultimately, β-casein gene expression (Akhtar et al., 2009). This link may be provided by basal localization and activation of PI3K downstream of laminin stimulation, which leads to Rac1 activation and sustained Stat5 tyrosine phosphorylation (Xu et al., 2010). The likely role for Rac1 to support Jak2-Stat5 signal relay is through decreased association of SHP-2 with Jak2 (Akhtar and Streuli, 2006).

MECs cultured on either plastic or collagen I are refractory to the stimulation of prolactin (Edwards et al., 1998). One mechanism to explain the defectiveness in prolactin signaling is that the ligand and receptor are physically segregated from each other. Prolactin receptor is basolaterally localized, while prolactin in the culture medium is delivered to the apical side of a 2D cell layer under these culture conditions. This is in contrast with 3D cultures, in which ligand can directly encounter the receptor on the basolateral surface of acini (Xu et al., 2009). However, interestingly, in sparse MEC monolayers, prolactin is still unable to activate its signaling pathway in cells located at the edge of epithelial islands (Streuli et al., 1995). This indicates that novel mechanisms are also involved in suppressing prolactin signaling in MECs cultured on 2D collagen substrata.

We have previously explored how cell adhesion controls other signaling pathways in MECs, and discovered that insulin signaling is under strict control of cell–matrix interactions (Farrelly et al., 1999; Lee and Streuli, 1999). In MECs cultured on plastic, RhoA is highly activated, and Rok stimulates the serine phosphorylation of IRS-1, hampering insulin-induced tyrosine phosphorylation of IRS-1 (Lee et al., 2009). Interestingly, Rok and myosin II confer “higher than normal” stiffness in 2D cultures of breast cells. Since cell stiffness inversely correlates with the ability of MECs to synthesize β-casein, we reasoned that activation of RhoA-Rok-myosin II pathway in 2D cultures might directly compromise prolactin signaling (Alcaraz et al., 2008).

In this study, we have therefore characterized the role of the RhoA-Rok-myosin II pathway in prolactin signaling. We demonstrate this pathway is deleterious to prolactin signaling, providing a further mechanism to explain how an abnormal tissue microenvironment (i.e., 2D) controls cell fate decisions in MECs.

## Materials and Methods

### Reagents

Bovine insulin, ovine prolactin, mouse EGF, and hydrocortisone were purchased from Sigma (St. Louis, MO). Antibodies to RhoA, Stat5, β-casein, Erk, and hemagglutinin (HA) were from Santa Cruz Biotechnology (Santa Cruz, CA). Rho activation assay kit, Rac activation assay kits, and antibody to phospho-Stat5 (Tyr694) were from Upstate Biotechnology (Lake Placid, NY). Y27632, blebbistatin, and ZVAD-fmk were obtained from Calbiochem (San Diego, CA).

### Substrata and cell culture

Collagen I- and laminin-coated dishes were prepared by incubating plates overnight at 4°C with rat tail collagen (Becton Dickinson, Bedford, MA) and laminin (Becton Dickinson) at 8 µg/cm^2^, respectively. The plates were washed with phosphate-buffered saline before use. Reconstituted BM matrix, Matrigel, was purchased from Becton Dickinson and coated onto dishes at 7 mg/ml.

All experiments were performed with first or second passage MECs derived from mid-pregnant ICR mice. Primary epithelial cultures were prepared from isolated mammary alveoli and plated on different substrata in nutrient mixture F-12 (Sigma) containing 10% fetal bovine serum (Hyclone), 1 mg/ml fetuin (Sigma), 5 ng/ml EGF, 5 µg/ml insulin, and 1 µg/ml hydrocortisone. After 72 h, medium was changed to Dulbecco's modified Eagle's medium (DMEM)/nutrient mixture F-12 (Invitrogen, Grand Island, NY) containing hydrocortisone and insulin, and cells were subjected to various treatments. To examine the effect of re-adhesion to ECM on prolactin receptor mRNA levels, cells were trypsinized and replated onto either collagen I or BM. In this study, animals were obtained, maintained, and used in accordance with the policies of the Institutional Animal Care and Use Committee of the Chung Shan Medical University.

### Adenovirus infection

Recombinant adenovirus carrying HA-tagged dominant negative RhoA (N19RhoA), constitutively active RhoA (L63RhoA), and dominant negative Rok (RB/PH (TT)) were generated as previously described (Ming et al., 2002). For cells cultured on collagen I, recombinant adenovirus was added to cells directly; whereas for those cultured on BM, cells were trypsinized, infected in suspension at 37°C for 1 h, and then plated onto Matrigel-coated dishes. After incubated with adenovirus for 18–24 h, cells were cultured in DMEM/F-12 containing hydrocortisone and insulin, and stimulated with prolactin (3 µg/ml).

### Western blot analysis

Cells were lysed in lysis buffer containing 50 mM Tris (pH 7.4), 150 mM NaCl, 2 mM EDTA, 1 mM Na_3_VO_4_, 10 mM NaF, 10 µg/ml aprotinin, 10 µg/ml leupeptin, 1 mM phenylmethylsulfonyl fluoride, and 1% Triton-100. Whole cell lysates were subjected to SDS-PAGE, transferred to PVDF membrane (NEN), and probed with antibodies to RhoA (2 µg/ml), Rac1 (2 µg/ml), phospho-Stat5 (Tyr694) (1:1000), Stat5 (0.5 µg/ml), β-casein (0.4 µg/ml), Erk (0.4 µg/ml), and HA (0.4 µg/ml). Proteins were visualized using an ECL kit (Cell Signaling Technology, Danvers, MA).

### Rho and Rac activity assay

Measurement of Rho and Rac activity was performed according to manufacturer's instructions (Upstate Biotechnology). Briefly, ∼1 mg of cleared cell lysates were incubated for 1 h at 4°C with GST-Rhotekin Rho-binding domain bound to glutathione-agarose beads to precipitate GTP-bound Rho, and ∼1.5 mg of cell lysates were incubated with GST-PAK-1 p21-binding domain bound to glutathione-agarose beads to precipitate GTP-bound Rac. Total lysates and precipitates were analyzed by western blotting using antibody to RhoA or Rac.

### Reverse transcription polymerase chain reaction (RT-PCR) and real-time RT-PCR

Total RNA was isolated using TRIzol (Invitrogen) according to the manufacturer's instructions. The first strand cDNA synthesis was carried out with ImProm-II™ reverse transcriptase (Promega, Madison, WI) at 42°C for 1 h followed by incubation at 99°C for 5 min to denature reverse transcriptase. Amplification consisted of a denaturation step of 30 sec at 94°C, an annealing step of 1 min at 61°C, and an extension step of 90 sec at 72°C. Amplification was performed in 26 cycles for prolactin receptor, 24 cycles for GAPDH, 23 cycles for β-casein (for MECs cultured on collagen I), and 15 cycles for β-casein (for MECs cultured on BM). The PCR primers used for mouse β-casein were 5′-ATG CCC CTC CTT AAC TCT GAA-3′ (forward) and 5′-GCA TGA TCC AAA GGT GAA AAG-3′ (reverse); mouse prolactin receptor were 5′-ATA CTG GAG TAG ATG GGG CCA GGA GAA ATC-3′ (forward) and 5′-CTT CCA TGA CCA GAG TCA CTG TCA GGA TCT-3′ (reverse); and mouse GAPDH were 5′-ACC ACA GTC CAT GCC ATC AC-3′ (forward) and 5′-TCC ACC ACC CTG TTG CTG TA-3′ (reverse).

For real-time RT-PCR, cDNA was synthesized using High Capacity RNA-to-cDNA kit (Applied Biosystems, Carlsbad, CA). qPCR reactions were carried out on a StepOnePlus™ Real-Time PCR System (Applied Biosystems) using TaqMan® gene expression master mix for amplification of prolactin receptor (assay ID Mm00599957_m1, Applied Biosystems). MAPK1 was used as the endogenous control (assay ID Mm00442479_m1, Applied Biosystems). Cycling conditions were as follows: 50°C for 2 min, 95°C for 10 min, followed by 40 cycles of amplification at 95°C for 15 sec, and 60°C for 1 min.

## Results

### MECs cultured on plastic and collagen I exhibit higher RhoA activity than those cultured on BM

Prolactin and insulin play important roles in mammary differentiation, and their signaling requires cell adhesion to BM. Our recent work has demonstrated that the RhoA-Rok pathway is greatly activated in MECs cultured on plastic as compared to those cultured on BM, and this results in downregulation of insulin signaling (Lee et al., 2009). In this study, we have investigated the possibility that the RhoA-Rok pathway also accounts for the ineffectiveness of prolactin signaling in cells that are devoid of proper adhesion to BM and cultured in an abnormal tissue microenvironment. In most of the experiments, primary MECs isolated directly from mice were cultured on dishes coated with collagen I, the major ECM component in the stromal compartment of mammary gland. The results were compared with those using MECs cultured as 3D acini on BM.

**Fig. 1:**
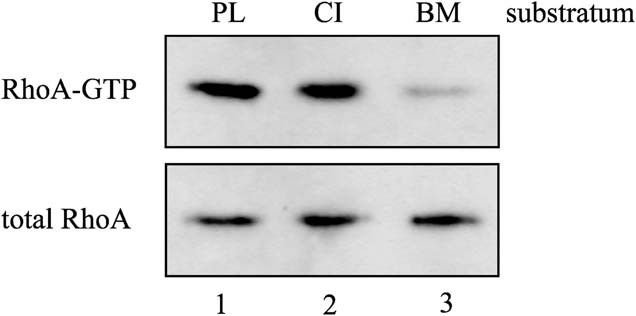
MECs cultured on plastic and collagen I exhibit higher RhoA activity than those cultured on BM. MECs were cultured on plastic (PL), collagen I (CI) or BM. Cell lysates were incubated with GST-Rhotekin Rho binding domain bound to glutathione-agarose beads to precipitate GTP-bound Rho. Total lysates and precipitates were then analyzed by immunoblotting using antibody to RhoA.

Initially, we examined RhoA activity in MECs cultured on plastic, collagen I, and BM. Our results showed that cells cultured on plastic and collagen I exhibited comparable levels of RhoA activity, which were much greater than those in cells cultured on BM (Fig. 1). This result indicates that ECM differentially regulates RhoA activity in MECs.

### Prolactin receptor is predominantly suppressed in MECs cultured on collagen I

**Fig. 2:**
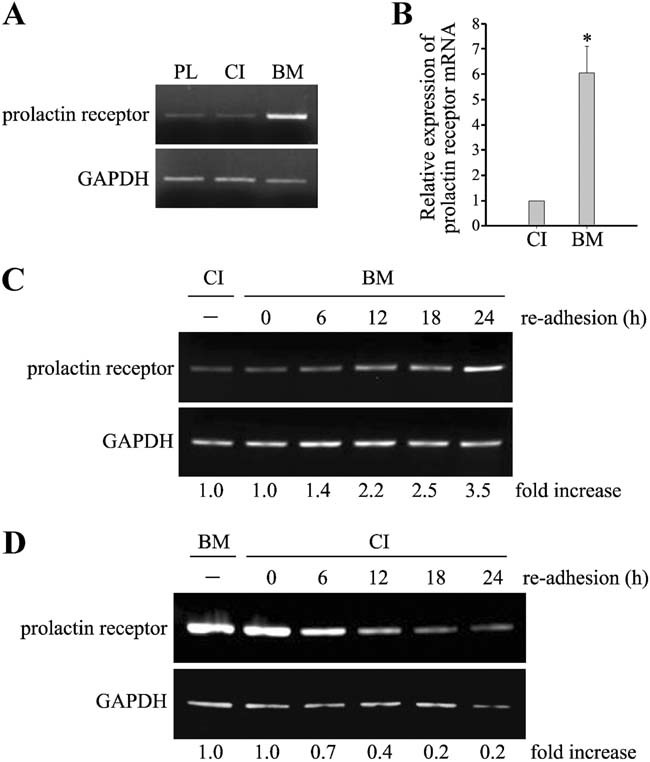
ECM regulates the expression of prolactin receptor. A: MECs were cultured on plastic (PL), collagen I (CI) or BM for 4 days. Total RNA was reverse transcribed and PCR-amplified with primers for prolactin receptor and GAPDH. B: MECs were cultured on collagen I or BM for 5 days. Total RNA was reverse transcribed and subjected to real-time PCR using primers for prolactin receptor and MAPK1. Relative expression of prolactin receptor normalized to MAPK1 is expressed as fold increase with respect to cells cultured on collagen I. *P < 0.05. C: MECs were either cultured on collagen I for 4 days, or cultured on collagen I for 3 days, then trypsinized, and replated on BM for 0–24 h. D: MECs were either cultured on BM for 4 days, or cultured on BM for 3 days, then trypsinized and replated on collagen I for 0–24 h. Total RNA was reverse transcribed and PCR-amplified with primers for prolactin receptor and GAPDH. Relative expression of prolactin receptor normalized to GAPDH is expressed as fold increase.

Our microarray results have indicated that expression of prolactin receptor mRNA is modulated by ECM (data not shown). We therefore measured the levels of prolactin receptor mRNA in MECs cultured on plastic, collagen I, and BM. In agreement with the microarray data, MECs cultured on BM displayed higher levels of prolactin receptor mRNA than those cultured on plastic and collagen I (Fig. 2A). The ECM dependence of prolactin receptor expression was confirmed by quantitative PCR (Fig. 2B).

To further confirm that the prolactin receptor mRNA levels were ECM-regulated, MECs were initially cultured on collagen I for 3 d, then trypsinized, and replated onto BM, or vice versa. We found that cell adhesion to BM always led to greater expression of prolactin receptor regardless the order of plating, and that the alteration of prolactin receptor mRNA in response to different ECM occurred within 6–12 h after cell plating (Fig. 2C,D).

These results suggest that ECM alone is able to control the expression of prolactin receptor, and this occurs within a few hours. Thus, modulation of prolactin receptor levels might represent a mechanism by which ECM influences the efficiency of prolactin signaling.

### RhoA exerts adverse effect on prolactin signaling in MECs

**Fig. 3:**
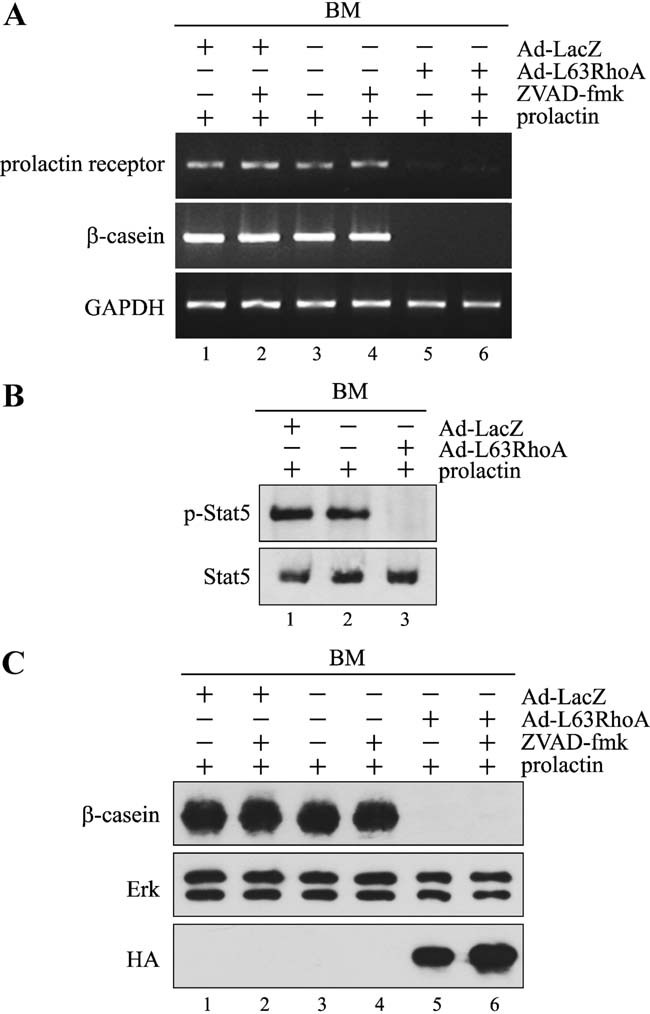
Constitutively active RhoA inhibits prolactin signaling in MECs cultured on BM. MECs cultured on BM were trypsinized, infected with adenovirus carrying HA-tagged constitutively active RhoA (Ad-L63RhoA) or LacZ (Ad-LacZ), and replated on BM for 24 h. A: Cells were then stimulated with 3 µg/ml prolactin in the absence or presence 100 µM ZVAD-fmk for 24 h. Total RNA was extracted, reverse transcribed, and PCR-amplified with primers for prolactin receptor, β-casein, and GAPDH. B: Cells were serum-starved for 8 h, and then stimulated with prolactin for 15 min. Cell lysates were analyzed by immunoblotting with antibodies to phospho-Stat5 (p-Stat5) and Stat5. C: Cells were stimulated with prolactin in the absence or presence ZVAD-fmk for 36 h. Total cell lysates were analyzed by immunoblotting with antibodies to β-casein, Erk, and HA. Levels of Erk were used as loading control.

Since RhoA inhibits insulin signaling in MECs, we reasoned that it might have a similar effect on prolactin signaling, either at the level of prolactin receptor expression or further downstream. We therefore expressed constitutively active RhoA (L63RhoA) in MECs cultured on BM. This diminished the expression of prolactin receptor, and completely prevented prolactin-induced Stat5 tyrosine phosphorylation and β-casein expression (Fig. 3). To confirm that RhoA-mediated inhibition of prolactin signaling is not secondary to the induction of apoptosis, ZVAD-fmk was added to the cultures to prevent caspase activation. ZVAD-fmk did not eliminate the inhibitory effect of RhoA on β-casein expression (Fig. 3). Thus, RhoA activation prevents prolactin receptor expression and Stat5 signaling.

**Fig. 4:**
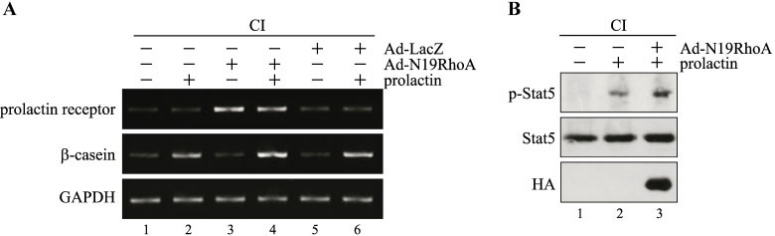
Expression of dominant negative RhoA improves prolactin signaling in MECs cultured on collagen I. MECs cultured on collagen I (CI) were infected with adenovirus carrying HA-tagged dominant negative RhoA (Ad-N19RhoA) or LacZ (Ad-LacZ) in situ for 24 h. A: Cells were then stimulated without or with 3 µg/ml prolactin for 24 h. Total RNA was extracted, reverse transcribed, and PCR-amplified with primers for prolactin receptor, β-casein, and GAPDH. Due to the low levels of β-casein expression in these cells, the number of cycles for PCR-amplification of β-casein cDNA was increased as described in Materials and Methods section. (B) Cells were serum-starved for 8 h, and then stimulated with prolactin for 15 min. Cell lysates were analyzed by immunoblotting with antibodies to phospho-Stat5 (p-Stat5), Stat5, and HA.

We also expressed dominant negative RhoA (N19RhoA) in MECs cultured on collagen I. Cells cultured on collagen I express very low levels of casein mRNA and Stat5 is phosphorylated to a low level, in comparison with the extent of prolactin signaling and casein expression in MECs on BM (Streuli and Bissell, 1990; Edwards et al., 1998). N19RhoA elevated the levels of prolactin receptor and prolactin-induced Stat5 phosphorylation in MECs cultured on collagen I (Fig. 4). However, interestingly, β-casein expression was not substantially increased (Fig. 4A and data not shown).

These results suggest that the low level of prolactin receptor expression in MECs cultured in 2D on collagen I is due to hyperactivation of RhoA.

### Suppression of Rok enhances prolactin signaling in MECs cultured on collagen I

**Fig. 5:**
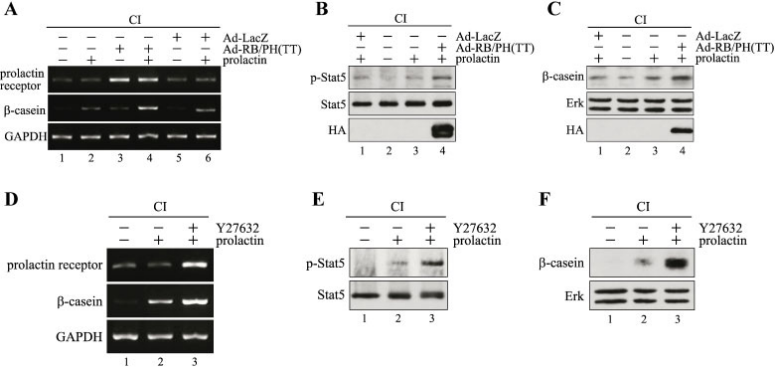
Expression of dominant negative Rok or application of Rok inhibitor Y27632 enhances prolactin signaling in MECs cultured on collagen I. A–C: MECs cultured on collagen I (CI) were infected with adenovirus carrying HA-tagged dominant negative Rok (Ad-RB/PH (TT)) or LacZ (Ad-LacZ) in situ for 24 h. D–F: MECs cultured on collagen I were pretreated with 10 µM Y27632. A,D: Cells were then stimulated without or with 3 µg/ml prolactin for 24 h. Total RNA was isolated, reverse transcribed, and PCR-amplified with primers for prolactin receptor, β-casein, and GAPDH. B,E: Cells were stimulated without or with prolactin for 15 min. Cell lysates were analyzed by immunoblotting with antibodies to phospho-Stat5 (p-Stat5), Stat5 or HA. C,F: Cells were stimulated without or with prolactin for 36 h. Total cell lysates were analyzed by immunoblotting with antibodies to β-casein, Erk, or HA.

Rok is a key effector of RhoA, and we therefore tested its involvement in the inhibition of prolactin signaling in MECs cultured on collagen I. For these experiments, we either added the Rok inhibitor, Y27632 to cultures, or expressed dominant negative Rok (RB/PH (TT)). In both cases, blocking the action of Rok enhanced prolactin receptor expression (Fig. 5A), as well as prolactin-induced Stat5 phosphorylation (Fig. 5B) and β-casein expression (Fig. 5A,C).

These results demonstrate that the RhoA-Rok pathway, which is activated in MECs cultured in 2D on collagen I, prevents prolactin signaling. Suppressing Rok restores the levels of prolactin receptor and permits prolactin-activated signaling.

### Inhibition of myosin II activity augments prolactin signaling in MECs cultured on collagen I

**Fig. 6:**
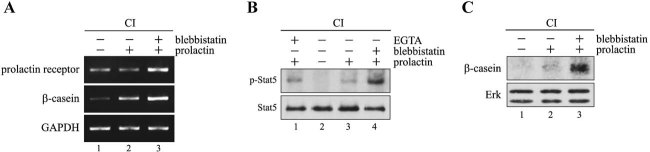
Blebbistatin augments prolactin signaling in MECs cultured on collagen I. A,C: MECs cultured on collagen I (CI) were pretreated without or with 25 µM blebbistatin for 1 h, and then stimulated with 3 µg/ml prolactin for 24 or 36 h. Total RNA was isolated after 24-h stimulation, reverse transcribed, and PCR-amplified with primers for prolactin receptor, β-casein, and GAPDH (A). Total cell lysates were prepared after 36-h stimulation, and analyzed by immunoblotting with antibodies to β-casein and Erk (C). B: MECs were untreated, pretreated with 25 µM blebbistatin for 12 h, or pretreated with 5 mM EGTA for 1 h, and then stimulated with prolactin for 15 min. Cell lysates were analyzed by immunoblotting with antibodies to phospho-Stat5 (p-Stat5) and Stat5.

An important function of Rok is to induce actomyosin contraction. To do so, Rok phosphorylates myosin light chain and myosin light chain phosphatase to enhance myosin II activity (Vicente-Manzanares et al., 2009). To determine whether myosin II acts downstream of Rok to impair prolactin signaling in cells cultured on collagen I, blebbistatin was used to inhibit non-muscle myosin II ATPase activity, and its effect on prolactin signaling was evaluated. Blebbistatin significantly augmented prolactin receptor expression, as well as prolactin-induced Stat5 phosphorylation and β-casein synthesis (Fig. 6).

Taken together, our results demonstrate that the failure of prolactin signaling in MECs cultured on collagen I is due to hyperactivation of the RhoA-Rok-myosin II pathway. Suppressing this pathway converts MECs to become more responsive to prolactin.

It has previously been suggested that MECs in 2D culture are refractory to prolactin stimulation because prolactin receptor is basolaterally localized and therefore topologically separated from its ligand (Xu et al., 2009). We performed similar experiments and found that EGTA treatment slightly improved prolactin-induced Stat5 phosphorylation, however there was a significantly greater induction of prolactin signaling in cells treated with blebbistatin (Fig. 6B).

These data suggest that the RhoA-Rok-myosin II pathway influences prolactin signaling through additional mechanisms that are distinct to altering the spatial distribution of prolactin receptor. We argue that modulation of prolactin receptor expression is also involved.

### Altering RhoA-Rok activity inversely affects Rac activity in MECs

**Fig. 7:**
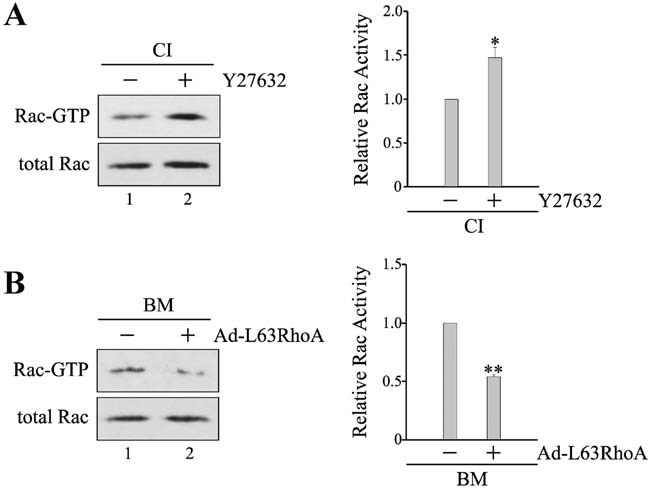
Application of Y27632 into MECs cultured on collagen I increases Rac activity, whereas expression of constitutively active RhoA in MECs cultured on BM decreases Rac activity. A: MECs cultured on collagen I (CI) were treated with 15 µM Y27632 for 12 h. B: MECs cultured on BM were trypsinized, mock-infected, or infected with adenovirus carrying constitutively active RhoA (Ad-L63RhoA), and replated on BM for 24 h. Cell lysates were incubated with GST-PAK1 p21-binding domain bound to glutathione-agarose beads to precipitate GTP-bound Rac. Total lysates and precipitates were then analyzed by immunoblotting using antibody to Rac. Immunoblots from three independent experiments were analyzed by densitometry. Relative Rac activity is indicated by the amount of Rac-GTP normalized to that of total Rac, and values are expressed as fold stimulation with respect to untreated cells or mock-infected cells. *P < 0.05; **P < 0.01.

The mutual antagonism between Rac and Rho GTPases has been well documented (Nimnual et al., 2003; Ohta et al., 2006; Takefuji et al., 2007; Bustos et al., 2008). Given that Rac1 is essential for optimal prolactin signaling in MECs, we asked if the effect of RhoA pathway on prolactin signaling was mediated by inhibiting Rac1 (Akhtar and Streuli, 2006). Rac activity was therefore assessed in response to alterations of RhoA and Rok activity. Inhibition of Rok by Y27632 resulted in elevated Rac activity in cells cultured on collagen I, while constitutively active L63RhoA reduced Rac activity in cells cultured on BM (Fig. 7).

These results show that the RhoA pathway has a negative impact on Rac activity in MECs, and that a blockade of this pathway leads to a rise in Rac activity. Thus, the effect of RhoA pathway on prolactin signaling might be, in part, through lowering Rac activity.

### MECs cultured on 2D laminin are ineffective in prolactin signaling

**Fig. 8:**
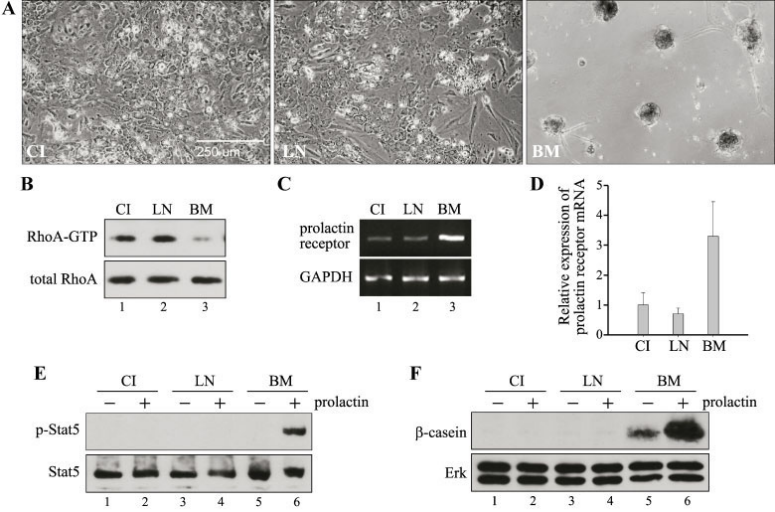
MECs cultured on 2D collagen I and 2D laminin are less effective in prolactin signaling than those cultured on 3D BM. MECs were cultured on 2D collagen I (CI), 2D laminin (LN), or 3D BM for 4 days. A: Phase contrast micrographs of the cells. B: Cell lysates were incubated with GST-Rhotekin Rho binding domain bound to glutathione-agarose beads to precipitate GTP-bound Rho. Total lysates and precipitates were then analyzed by immunoblotting using antibody to RhoA. C: Total RNA was reverse transcribed and PCR-amplified with primers for prolactin receptor and GAPDH. D: Total RNA was reverse transcribed and subjected to real-time PCR using primers for prolactin receptor and MAPK1. Relative expression of prolactin receptor normalized to MAPK1 is expressed as fold increase with respect to cells cultured on collagen I (n = 3). E: Cells were serum-starved for 8 h, and then stimulated without or with 3 µg/ml prolactin for 15 min. Cell lysates were analyzed by immunoblotting with antibodies to phospho-Stat5 (p-Stat5) and Stat5. F: Cells were stimulated without or with prolactin for 36 h. Total cell lysates were analyzed by immunoblotting with antibodies to β-casein and Erk.

Our results show that prolactin signaling in MECs cultured on the soft BM hydrogel is more efficient than signaling in cells cultured on the rigid surface of collagen I-coated plastic. These culture conditions differ in both matrix rigidity and composition. Since laminin is the major component of BM and is essential for prolactin signaling, we examined prolactin signaling in MECs cultured on laminin-coated plastic, which has about the same rigidity as collagen I-coated dishes. This would help to show whether matrix stiffness affects prolactin signaling. Similar to MECs cultured on 2D collagen I, cells on 2D laminin formed monolayers, and exhibited higher RhoA activity than those cultured on 3D BM (Fig. 8A,B).

The cells on 2D laminin were also less responsive to prolactin stimulation than cells on 3D BM. We found that the levels of prolactin receptor, Stat5 tyrosine phosphorylation, and β-casein expression were comparable to those in cells cultured on 2D collagen I, but were far less than in cells on 3D BM (Fig. 8C–F).

These data indicate that matrix rigidity substantially influences prolactin signaling in MECs by regulating the levels of prolactin receptor. Thus, both the type and texture of matrix are crucial determinants of optimal prolactin signaling.

## Discussion

### The RhoA pathway prevents mammary differentiation

Mammary epithelia contact BM in vivo, and culturing MECs in vitro on a reconstituted BM matrix reestablishes the differentiated phenotype. In contrast, culturing MECs on a thin-layer of collagen I renders prolactin signaling ineffective (Edwards et al., 1998). Here we demonstrate that the failure of prolactin signaling in cells on collagen I results from enhanced activation of the RhoA-Rok-myosin II pathway. We discovered that suppressing this pathway by blocking RhoA, Rok, or myosin II ATPase elevates the levels of prolactin receptor and improves prolactin signaling. Conversely, activating this pathway in MECs cultured on BM by overexpressing RhoA, suppresses prolactin receptor expression and milk protein gene expression.

Insulin augments prolactin signaling to stimulate maximal levels of β-casein expression, and we have previously discovered that the RhoA-Rok pathway also prevents insulin signaling (Lee et al., 2009). However, the mechanisms for inhibiting insulin and prolactin signaling are different. In the case of insulin signaling, Rok directly binds and inhibits IRS-1 tyrosine phosphorylation. Interestingly, constitutively active PKN, a downstream effector of RhoA, impairs tight junction sealing in MECs by blocking the signaling of another lactogenic hormone, glucocorticoid (Fischer et al., 2007).

These studies collectively imply that the RhoA pathway is detrimental to the signaling of lactogenic hormones. RhoA therefore compromises the differentiated function of mammary glands. Our new data provide a novel mechanism to explain why MECs don't differentiate when they are cultured on 2D collagen substrata.

### Prolactin receptor expression

We have found that primary MECs cultured on collagen I have lower amounts of prolactin receptor than those on BM, correlating with their poor responses to prolactin stimulation. Interestingly, this contradicts with other observations that showed no differences in levels of prolactin receptor in mammary EpH4 cells cultured on plastic with and without a BM overlay (Xu et al., 2009). We suggest that the experimental conditions, i.e., culturing cells on top of BM in our system, versus plating cells on plastic and overlaying them with BM in the EpH4 study, causes the discrepancy. However, our conclusions are supported by experiments in Src knockout mice, which display decreased expression of prolactin receptor in mammary glands (Watkin et al., 2008). Src phosphorylates and activates p190RhoGAP; so deleting Src might result in elevated RhoA activity, thereby reducing the expression of prolactin receptor (Arthur et al., 2000).

It is currently unclear how signals from the ECM alter prolactin receptor expression. Our data indicate that this occurs via modulation of the RhoA-Rok-myosin II pathway, but the downstream mechanisms are not elucidated yet. One possibility is that ECM controls the levels or activity of the transcription factors that are involved in prolactin receptor expression. These transcription factors are C/EBPβ, Sp1/Sp3, and estrogen receptor α (ERα) (Hu et al., 1998; Dong et al., 2006). Intriguingly, both C/EBPβ and ERα expression were found to be higher in cells cultured on BM than those on plastic ((Novaro et al., 2003) and data not shown). This ECM-dependent expression pattern for transcription factors may well confer higher levels of prolactin receptor in MECs cultured on BM.

### RhoA pathway and cell stiffness

In response to ECM adhesion, cells probe the rigidity of microenvironment, and respond it by exerting traction forces (Discher et al., 2005; Chen, 2008). This force is generated by activating the RhoA-Rok-myosin II pathway, giving rise to actomysoin contraction (Wozniak et al., 2003; Paszek et al., 2005). The tension on cytoskeleton consequently influences cell stiffness (Schedin and Keely, 2011).

For the mammary cell lines EpH4 and SCp2, cells cultured on 2D rigid surfaces are stiffer than a normal mammary organoids, and inhibiting either Rok or myosin II activity decreases cell stiffness (Alcaraz et al., 2008). Such cellular elasticity has a close link with differentiation, because when it deviates from the normal range, MECs lose the ability to express β-casein.

Our results are in agreement with this finding. In MECs cultured on thin, 2D layers of collagen I or laminin, RhoA activity is high, and β-casein expression is suppressed. Under this cell–matrix configuration, which normally leads to high cytoskeletal tension and low elasticity, cells become responsive to prolactin only when the RhoA-Rok-myosin II pathway is blocked and the internal tension is released. We suggest that high ECM rigidity and aberrant activation of RhoA jeopardizes mammary cell differentiation by altering the cell's elasticity, and thereby preventing transmission of differentiation signals.

### Tension signaling and gene expression

How ECM rigidity, and the resulting cell stiffness, regulate gene expression is not completely understood. The connections between the cytoskeleton and the nucleus are hard-wired via the linker of nucleoskeleton and cytoskeleton (LINC) complex. Thus, mechanical signals derived from cell–ECM interactions are transmitted through these scaffolds and sent into the nucleus, leading to alterations in gene transcription (Spencer et al., 2007; Wang et al., 2009).

A crucial aspect for this type of mechanotransduction is that, in order to propagate mechanical signals, the cytoskeleton needs to be tensed (Hu et al., 2005). This brings about the possible involvement of the RhoA-Rok-myosin II pathway in modulation of gene expression as it induces cytoskeletal tension. In this regard, the RhoA pathway could have a rather direct effect on gene expression. Whether β-casein or prolactin receptor genes are regulated by this manner merits further investigation.

### Rho-Rac antagonism

One further possibility for RhoA to inhibit prolactin signaling is to antagonize Rac1, which is required mammary differentiation (Akhtar and Streuli, 2006). RhoA suppresses Rac activity via Rok by either activating Rac GAP or downregulating the function of Rac GEF (Ohta et al., 2006; Takefuji et al., 2007). A recent finding further shows that myosin II binds and inhibits Dbl family GEF, resulting in the inhibition of Rac activity (Lee et al., 2010).

Rho and Rac are normally balanced, but overactivation of RhoA would suppress Rac, thereby hampering prolactin signaling, as we observed in this study. Conversely, suppressing Rok or myosin II elevates Rac activity, converting cells into a state that is more permissive for prolactin signaling. We suggest that, in addition to its effects on cytoskeletal tension, the RhoA pathway inhibits prolactin signaling by antagonizing Rac activity. Thus RhoA may inhibit differentiation at two levels: first through a tension-mediated regulation of prolactin receptor levels, and second through a Rac-dependent control on prolactin signaling downstream of the receptor.

## Summary

Within a tissue, cells constantly monitor their microenvironment by interacting with ECM. This external stimulus is transmitted inward via a variety of biochemical and mechanical signals, and is interpreted by reprogramming gene expression. RhoA stands in a key position in the process of interpreting the cellular microenvironment and propagating intracellular signals to control cell fate decisions.

Under the physiological conditions such as lactation, MECs exhibit basal levels of RhoA activity to maintain their morphology and differentiated phenotype. On the other hand, in some pathological states whereby the type or texture of ECM is altered, such as fibrosis or neoplasia, RhoA activity is correspondingly increased, skewing cell behavior away from differentiation, and towards proliferation and migration (Butcher et al., 2009).
